# Supplementary material for: Identification of photoperiod-induced specific miRNAs in the adrenal glands of Sunite sheep (Ovis aries)
Source: Front Vet Sci. 2022 Jul 22;9:888207. doi: 10.3389/fvets.2022.888207 (PMC9354845; doi:10.3389/fvets.2022.888207)
Supplement: Supplementary file 1 [file Data_Sheet_1.ZIP › Supplementary file/miRDeep2 file.zip!/miRDeep2 file/Novel_Structure/SP42A1/Novel_113.pdf]

Provisional ID	:	NC_019460.2_7414
Score total	:	33.9
Score for star read(s)	:	3.9
Score for read counts	:	26.6
Score for mfe	:	1.8
Score for randfold	:	1.6
Score for cons. seed	:	
Total read count	:	64
Mature read count	:	63
Loop read count	:	0
Star read count	:	1

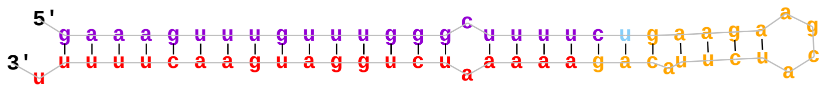

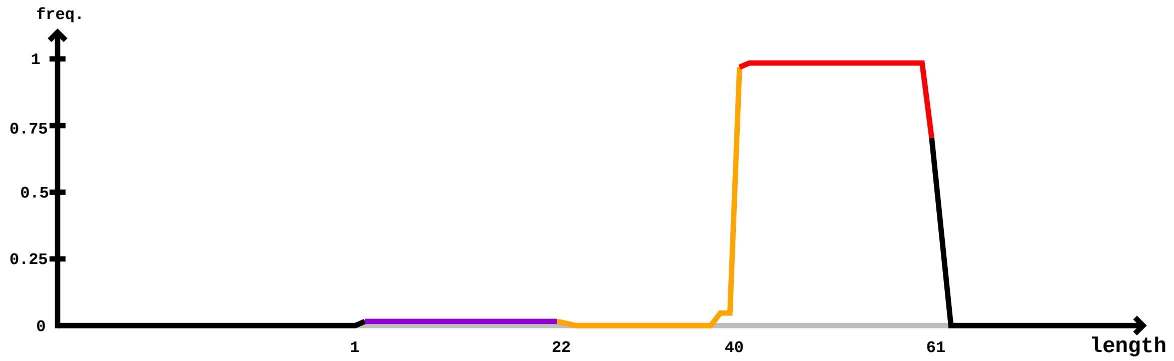

Star **Mature**

```
5'- ugaaaaacuuugauuauacugaguuggccagaaaguuuguuugggcuuuucugaagaagcaucuuacagaaaaaucuggaugaacuuuuuggccaauccaacaauauauuu   -3'   obs
    ugaaaaacuuugauuauacugaguuggccagaaaguuuguuugggcuuuucugaagaagcaucuuacagaaaaaucuggaugaacuuuuuggccaauccaacaauauauuu         exp
    ...................((.(((((((((((((((((((((((.(((((((((((....)))).))))))).))))))))))))))))))))))).))...........   reads   mm   sample
    ..............................gaaaguuuguuugggUuuuuc............................................................   1       1    seq
    ...................................................................agaaaaaucugAaugaacuuuu......................   1       1    seq
    ...................................................................agaaaaaucugAaugaacuuuuu.....................   2       1    seq
    .....................................................................aaaaaucugAaugaacuuuu......................   17      1    seq
    .....................................................................aaaaaucugAaugaacuuuuu.....................   42      1    seq
    ......................................................................aaaaucuggaugaacuuuuug....................   1       0    seq
```
